# Changes in attitudes towards hastened death among Finnish physicians over the past sixteen years

**DOI:** 10.1186/s12910-018-0290-5

**Published:** 2018-05-30

**Authors:** Reetta P. Piili, Riina Metsänoja, Heikki Hinkka, Pirkko-Liisa I. Kellokumpu-Lehtinen, Juho T. Lehto

**Affiliations:** 1 Faculty of Medicine and Life Sciences, University of Tampere, Tampere, Finland; 2 Department of Oncology, Palliative Care Unit, Tampere University Hospital, Teiskontie 35, R-building, 33520 Tampere, Finland; 3 Faculty of Social Sciences, University of Tampere, Tampere, Finland; 4 Rehabilitation Center Apila, Kangasala, Finland

**Keywords:** Clinical ethics, Decision-making, End-of-life care, Euthanasia

## Abstract

**Background:**

The ethics of hastened death are complex. Studies on physicians’ opinions about assisted dying (euthanasia or assisted suicide) exist, but changes in physicians’ attitudes towards hastened death in clinical decision-making and the background factors explaining this remain unclear.

The aim of this study was to explore the changes in these attitudes among Finnish physicians.

**Methods:**

A questionnaire including hypothetical patient scenarios was sent to 1182 and 1258 Finnish physicians in 1999 and 2015, respectively. Two scenarios of patients with advanced cancer were presented: one requesting an increase in his morphine dose to a potentially lethal level and another suffering a cardiac arrest. Physicians’ attitudes towards assisted death, life values and other background factors were queried as well. The response rate was 56%.

**Results:**

The morphine dose was increased by 25% and 34% of the physicians in 1999 and 2015, respectively (*p* < 0.001). Oncologists approved the increase most infrequently without a significant change between the study years (15% vs. 17%, *p* = 0.689). Oncological specialty, faith in God, female gender and younger age were independent factors associated with the reluctance to increase the morphine dose. Euthanasia, but not assisted suicide, was considered less reprehensible in 2015 (*p* = 0.008). In both years, most physicians (84%) withheld cardiopulmonary resuscitation.

**Conclusion:**

Finnish physicians accepted the risk of hastening death more often in 2015 than in 1999. The physicians’ specialty and many other background factors influenced this acceptance. They also regarded euthanasia as less reprehensible now than they did 16 years ago.

## Background

Discussions about the ethical justification of hastened death due to unbearable suffering are ongoing. Assisted death through euthanasia or physician-assisted suicide (PAS) has been legalized in seven countries (five states in the United States of America) thus far [1]. In addition, public support for euthanasia and PAS is mounting all over Western Europe, while some decline has been observed in the United States of America and Eastern Europe [1, 2]. Today, there are debates about the legalization of euthanasia in many countries, including Finland, where the government is currently considering options after a civil motion demanding the legalization of euthanasia. At the same time, the importance of palliative care and patient-centred decision- making has been increasingly recognized among health care professionals and the general public in European countries including Finland [3–9].

Palliative care, by definition, intends to neither hasten nor postpone death [10]. The International Association for Hospice and Palliative Care and the European Association for Palliative Care have recently stated that euthanasia and PAS should not be included as part of the clinical practice of palliative care [11, 12]. Attitudes among physicians towards assisted death are not widely studied, but several surveys do demonstrate a lower amount of support from physicians for euthanasia and PAS when compared to support from the general public [1].

Questions about hastening death in end-of-life care are complex and may include ethical concerns broader than just euthanasia or PAS. Although clear definitions have been specified for euthanasia and PAS [12], which lead to a clearly assisted death, the term “hastened death” is unspecified and has many interpretations. The termination of life-sustaining treatments may be confused with euthanasia and PAS among the public and physicians [13]. The term “double effect” has been used when the act intended to do good (e.g., relief of suffering) justifies the foreseeable danger of harm (e.g., hastened death) [14]. The use and dosing of opioids during end-of-life care is a commonly used example when talking about the double effect: does the intent to treat pain or breathlessness outweigh the risk of potentially hastening death [15, 16]?

However, there is growing evidence that even though high doses of opioids may cause respiratory depression [17–19], they do not seem to hasten death during end-of-life care [20, 21]. In a large multinational study by Miccinesi et al., there was general approval for alleviating symptoms with a possibly life-shortening treatment [22]. In another study from the United Kingdom (UK), physicians reported that they had at least some intention to hasten death in 7.4% of the deaths evaluated [23]. Physicians’ attitudes towards hastened death through a dual effect and the background factors influencing these decisions remain largely unknown.

The aims of our study were to elucidate how, if at all, the attitudes and values towards assisted death among Finnish physicians have changed over the past 16 years and to determine the attitudes and background factors affecting physicians’ willingness to accept hastened death in a hypothetical patient scenario.

## Methods

### Participants

A postal survey was conducted in spring 1999 and in autumn 2015. In both years, the questionnaire was sent to 500 general practitioners (GPs), 300 surgeons, and 300 internists randomly selected from the register of the Finnish Medical Association and to all Finnish oncologists (*n* = 82 in 1999 and *n* = 158 in 2015). Reminders were sent twice to non-respondents. A cover letter including an introduction to the study and an assurance of anonymity was mailed together with the questionnaire. It was also stated in the cover letter, that answering to the questionnaire was completely voluntary. This study was approved by the Regional Ethics Committee of Tampere University Hospital, Finland (R15101).

### Questionnaire

The questionnaire included seven hypothetical patient scenarios. Following the patient scenarios, attitudes regarding several moral and ethical aspects were assessed with a 100-mm visual analogue scale (VAS) from “definitely agree” (0 mm) to “definitely disagree” (100 mm). These included, for example, statements concerning euthanasia, palliative care, the role of religion in ethical decisions, advanced care directives and health care economics together with physicians’ satisfaction with their own health, work and salary (Tables 2 and 3). Physicians’ personal conceptions of professional status and their own health, family life, religion, and nature and standard of living were assessed using a four-point Likert scale (Table 2). The questionnaire has been previously used and validated with Finnish physicians [24–26].

### Patient scenarios

In this study, we included two patient scenarios:

- In scenario 1, a 60-year-old male patient is suffering from prostatic cancer with metastases. Metastases in the thoracic spine led to total paraparesis 1 month earlier. There is no hope for a cure. The patient is well aware of the situation. He has totally lost his will to live. When you are together with him alone, he asks for a sufficient dose of morphine to “get away”. You have denied the overdose, explaining that it is against your ethical principles. During the following days, you notice that the patient asks you to double his morphine dose because of unbearable pain. The anti-inflammatory pain medication is at its maximum dose and you suspect if the pain is real (this sentence was removed from the scenario in 2015 as it did not comply with current treatment guidelines for cancer pain). You suppose that increasing the dose in such a way would lead to the patient’s death. Your decision is which of the following: a) to raise the dose because the patient has the right to sufficient pain relief in this end-of-life (terminal) care situation; b) to try to help the patient in other ways, such as with antidepressants, thus continuing with morphine dosing according to given guidelines; c) I can’t say; or d) give another solution:_______________________.

- In scenario 2, a 32-year-old female patient is brought by ambulance to the emergency unit. She is accompanied by her husband who says his wife has inoperable brain cancer. She has been receiving maximum radiotherapy, but this was discontinued 3 weeks ago. She has deteriorated considerably during the past week. The patient has now had an epileptic seizure and has been unconscious since the attack. After 20 min at the hospital the patient stops breathing, and there is no pulse. Your treatment decision is which of the following: a) to start cardiopulmonary resuscitation (CPR) or b) to withhold CPR.

### Statistical analysis

The answers concerning the doubling of the morphine dose in scenario 1 were converted to two options: “I accept” (response a) and “I do not accept” (other solutions). The answers on the 4- point Likert scale concerning values were converted to the following 2-point scale: 1–2 for “not important” and 3–4 for “important”.

Two-scale background factors and values were tested using the Pearson chi-square test.

Continuous variables were tested using an independent-variables *t*-test or the Mann-Whitney U- test if the data were not normally distributed. Two-sided *p*-values less than 0.05 were considered as statistically significant.

#### Logistic regression analysis

A forward stepwise logistic regression was used to create a model explaining the decision to increase the morphine dose. Background factors, life values, and attitudes, shown in Table 2, were all included in the model. The *p*-value limit for significance was set at 0.10 to enter and 0.15 to remove from the model.

Data analyses were performed using IBM SPSS Statistics for Windows, Version 23.0 (Armonk, NY: IBM Corp. Released 2014).

## Results

In total, 1373 valid responses were received (response rate 56%). Characteristics of the physicians according to the year of response are shown in Table 1. Compared to respondents in 1999, respondents in 2015 were older (*p* < 0.001), had longer working experience (*p* < 0.001) and were more often women (*p* < 0.001).

**Table 1:** Characteristics of the participants

	Surgeons				Internists				GPs				Oncologists				Total			
	1999		2015		1999		2015		1999		2015		1999		2015		1999		2015	
Number (% of total)	175	(24)	142	(22)	184	(25)	153	(24)	316	(43)	245	(38)	54	(7)	104	(16)	729	(100)	644	(100)
Response rate, %		58		47		61		47		63		49		51		66		62		51
Female, n (%)	33	(19)	47	(33)	60	(33)	81	(53)	170	(55)	173	(71)	30	(56)	85	(82)	293	(41)	386	(60)
Mean age (range)	48	(33–66)	51	(33–64)	48	(32–70)	52	(33–65)	42	(25–63)	47	(25–65)	46	(35–61)	48	(32–67)	45	(25–70)	50	(25–67)
Working place^a^																				
Outpatient unit	1	(1)	2	(1)	15	(9)	15	(10)	242	(78)	208	(86)	2	(4)	4	(4)	260	(37)	229	(36)
Hospital	146	(85)	124	(88)	123	(71)	122	(82)	33	(11)	24	(10)	44	(83)	91	(88)	346	(49)	361	(57)
Other	24	(14)	15	(11)	35	(20)	12	(8)	35	(11)	10	(4)	7	(13)	8	(8)	101	(14)	45	(7)
Years from graduation, median (range)^b^	22	(2–42)	26	(7–42)	21	(7–41)	26	(8–42)	16	(1–35)	21	(0–40)	18	(9–34)	22	(7–40)	19	(1–42)	23	(0–42)
Married, n (%)	140	(81)	119	(84)	142	(79)	124	(81)	228	(73)	198	(81)	45	(83)	71	(71)	555	(77)	512	(80)

^a^For 32 participants working place was not available

^b^For 19 participants year of graduation was not available

*GP* general practitioner

### Change in attitudes

The attitudes, personal factors and life values of the responding physicians in 1999 and 2015 are shown in Table 2.

**Table 2:** Attitudes, background factors and life values of the physicians in 1999 and 2015

	1999		2015		*P*-values*
Attitudes, median VAS (IQR)					
Active euthanasia is reprehensible	17	(6–51)	25	(5–66)	0.008
Withdrawal of life-sustaining treatments is reprehensible	89	(76–95)	93	(76–99)	< 0.001
Assisted suicide is reprehensible	14	(5–38)	13	(2–52)	0.480
End-of-life care is satisfying	36	(19–52)	15	(3–35)	< 0.001
People should pay costs of factitious diseases by themselves	44	(27–72)	78	(46–93)	< 0.001
Advance directives have been helpful in my decisions	35	(14–54)	10	(2–29)	< 0.001
Good palliative care enables good death	17	(9–28)	4	(1–12)	< 0.001
Physicians can’t estimate cancer pain	40	(25–70)	47	(27–72)	0.042
Religion has influence when I make ethical decisions	65	(31–93)	81	(47–98)	< 0.001
Being a doctor gives me satisfaction	20	(11–30)	7	(2–18)	< 0.001
My health is excellent	20	(10–32)	14	(6–26)	< 0.001
I feel burn out, tired to work	84	(63–94)	89	(71–97)	< 0.001
I’m pleased with my salary	72	(37–87)	22	(7–50)	< 0.001
It is waste of resources to treat patients > 80 years in ICU	73	(49–86)	77	(54–93)	< 0.001
Background factors and life values, n (%)					
Having children	600	(85)	555	(88)	0.057
Having own advance directive	38	(5)	38	(6)	0.668
Taking care of end-of-life patients in practice (last 2 years)	529	(75)	418	(65)	< 0.001
Taking care of a family member in end-of-life	513	(73)	314	(49)	< 0.001
Being afraid of death (Fear-of-death index)	580	(80)	544	(86)	0.006
Length of life is important	412	(59)	524	(87)	< 0.001
Health is important	711	(99)	610	(99)	0.027
Family is important	686	(95)	607	(99)	< 0.001
Clean environment is important	666	(93)	599	(98)	< 0.001
High standard of living is important	358	(50)	398	(65)	< 0.001
Faith in God is important	338	(48)	253	(42)	0.024
Success in professional career is important	639	(89)	377	(62)	< 0.001

*VAS* visual analogue scale

*IQR* interquartile range

*ICU* intensive care unit

*Mann-Whitney u-test for attitudes and Pearson Chi-Square for background factors and life values

Attitudes are expressed on a visual analogue scale (VAS) from 0 mm (definitely agree) to 100 mm (definitely disagree)

Euthanasia and withdrawal of life-sustaining treatments were considered slightly less reprehensible in 2015 than in 1999, whereas attitudes towards assisted suicide did not change significantly. In 2015, physicians more often believed that good palliative care enables a good death and found end-of-life care satisfying, although they were less often actually involved in end-of-life care than the respondents in 1999. Advance directives were considered more helpful in 2015, although physicians still rarely had their own advance directives. The impact of physicians’ background factors, faith in God, and religion on ethical decisions decreased between 1999 and 2015. The length of life, family, and cleanliness of environment were thought to be more important in 2015, while success in their professional career was less important.

### Change in decision-making

**Fig. 1:**
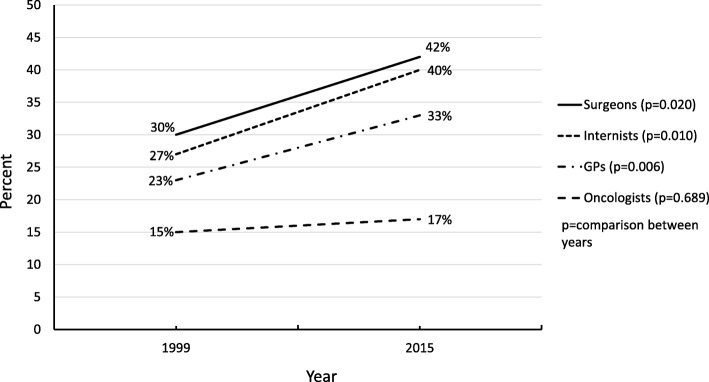
Proportion of respondents who were willing to increase the morphine dose among different physician groups in 1999 and 2015

In the case in scenario 1, physicians were significantly more willing to increase the morphine dose in 2015 (*n* = 219, 34%) than in 1999 (*n* = 180, 25%) (*p* < 0.001). This willingness increased in all groups of physicians, except among oncologists, who were also the most unwilling to do this in both years (Fig. 1). In contrast, 84% of the physicians decided to withhold CPR in case scenario 2 in both years. There were no significant changes regarding this decision about CPR among the different physician groups between the study years.

### Factors associated with physicians’ willingness to increase the morphine dose

Difference in the attitudes of physicians who accepted and those who did not accept the doubling of the morphine dose in both years studied are shown in Table 3.

**Table 3:** Attitudes of physicians who were willing or unwilling to increase the morphine dose in 1999 and 2015

	1999 Increasing the morphine dose					2015 Increasing the morphine dose				
Attitudes, median VAS (IQR)	Yes		No		*P*-value*	Yes		No		*P*-value*
Active euthanasia is reprehensible	37	(11–69)	14	(5–39)	< 0.001	33	(5–72)	24	(4–64)	0.162
Withdrawal of life-sustaining treatments is reprehensible	92	(83–96)	88	(72–95)	0.003	95	(80–99)	93	(75–98)	0.133
Assisted suicide is reprehensible	27	(9–61)	11	(4–28)	< 0.001	28	(2–68)	10	(1–49)	< 0.001
End-of-life care is satisfying	38	(19–51)	34	(18–52)	0.683	16	(2–30)	14	(3–45)	0.363
People should pay costs of factitious diseases by themselves	40	(23–65)	47	(29–75)	0.047	78	(49–94)	77	(47–93)	0.341
Advance directives have been helpful in my decisions	31	(10–55)	36	(15–54)	0.199	12	(2–29)	10	(2–28)	0.723
Good palliative care enables good death	19	(10–25)	16	(9–29)	0.833	4	(1–13)	4	(1–13)	0.869
Physicians can’t estimate cancer pain	35	(22–70)	41	(27–71)	0.056	44	(23–69)	50	(29–74)	0.006
Religion has influence when I make ethical decisions	77	(44–94)	57	(30–92)	0.040	86	(49–98)	78	(45–98)	0.130
Being a doctor gives me satisfaction	21	(11–29)	19	(11–30)	0.456	7	(1–19)	7	(2–18)	0.928
My health is excellent	21	(10–35)	20	(10–31)	0.273	15	(5–27)	14	(06–25)	0.751
I feel burn out, tired to work	84	(68–93)	84	(62–94)	0.701	88	(75–96)	89	(70–97)	0.843
I’m pleased with my salary	77	(51–90)	70	(35–87)	0.082	22	(8–50)	22	(7–51)	0.759
It is waste of resources to treat patients over 80 years of age in ICU	70	(48–86)	73	(49–87)	0.262	82	(54–94)	75	(54–91)	0.107

*VAS* visual analogue scale

*IQR* interquartile range

*ICU* intensive care unit

*Mann-Whitney U-test

Attitudes expressed on a visual analogue scale (VAS) from 0 mm (definitely agree) to 100 mm (definitely disagree)

In 1999, leniency towards euthanasia and assisted suicide was significantly greater in those who accepted the dose increase, while this was true only for assisted suicide in 2015.

Religion had a significantly larger influence on decision-making in physicians who accepted the morphine dose increase in 1999 but not in 2015.

Factors and attitudes that independently influenced physicians’ willingness to increase the morphine dose from the logistic regression analysis are shown in Table 4.

**Table 4:** Different background factors, life values and attitudes explaining physicians’ decision to increase the morphine dose (*n* = 323) versus not (*n* = 767) in forward logistic regression analysis

	*n*	*OR*	*(95% CI)*	*P-value*
Year of the survey				
1999	578	ref.		
2015	512	1.40	(1.05, 1.88)	0.024
Sex				
Female	534	ref.		
Male	556	1.51	(1.11, 2.05)	0.009
Age	1090	1.02	(1.01, 1.04)	0.007
Own advance directive				
No	1026	ref.		
Yes	64	1.74	(1.00, 3.03)	0.051
Faith in God				
Important	489	ref.		
Not important	601	1.64	(1.23, 2.19)	0.001
Assisted suicide is reprehensible (VAS)	1090	1.13	(1.08, 1.19)	< 0.001
Physicians can’t estimate cancer pain (VAS)	1090	0.94	(0.89, 0.99)	0.021
Physician groups				0.014
Oncologists	120	ref.		
Surgeons	252	2.50	(1.40, 4.46)	0.002
Internists	268	2.37	(1.34, 4.20)	0.003
GPs	450	2.30	(1.33, 3.97)	0.003

ref, reference

*VAS*, visual analogue scale

*GP*, general practitioner

Not being an oncologist was the most striking factor associated with physicians’ willingness to increase the morphine dose. In addition, physicians who were male, were older, did not believe in God, accepted assisted suicide, had doubts about physicians’ ability to assess cancer pain, and responded in 2015 were also more likely to be willing to increase the morphine dose. However, physicians’ decisions about CPR for the patient in scenario 2 and their attitudes towards euthanasia or withdrawal of life-sustaining treatments did not influence their decision to accept the escalation of the morphine dose.

## Discussion

Our study shows that some Finnish physicians’ attitudes and life values have changed substantially during the last 16 years. Their approval of euthanasia has slightly increased, whereas their acceptance of physician-assisted suicide (PAS) has remained low. In an end-of-life patient case scenario, physicians show an increasing willingness to give a high morphine dose, which might potentially hasten death. In logistic regression analysis, not being an oncologist, being male, and not believing in God were the most important background factors associated with physicians’ willingness to increase the morphine dose.

In our study Finnish physicians were less opposed to euthanasia now than they were 16 years ago. This finding is in agreement with previous studies showing increased acceptance of euthanasia in Europe as well as in Finland [1, 2]. However, attitudes towards euthanasia were measured with a continuous visual analogue scale (VAS) on a scale from 0 mm (reprehensible) to 100 mm (not reprehensible) in our study rather than with a dichotomous question (i.e., if the physician accepts or does not accept euthanasia).

Although using a VAS scale might have caused some confusion, doctors are generally familiar with its use. This type of assessment might provide more appropriate insight into this complex ethical question. Of note, the VAS median value in 2015 was still only 25 mm and the absolute difference compared to the value in 1999 was 8 mm. Thus, our results highlight the controversial attitude towards euthanasia, which might not be found in earlier studies; for example, a previous study showed that 46% of Finnish physicians supported legalization of euthanasia [2].

In contrast to other studies [1], Finnish physicians considered PAS even more reprehensible than euthanasia and this has not changed at all during the past 16 years. Determining whether this somewhat conflicting result is due to a true difference in the attitudes towards these two procedures or just less knowledge about the process of PAS in Finland could be an aim of future research.

We asked if euthanasia or assisted suicide were reprehensible or not with a VAS scale to evaluate the personal ethical attitudes of the physicians rather than their opinions on the general justification of these issues. Therefore, our results represent somewhat different aspect of these issues compared to the findings of studies that inquired about physicians’ opinions on the legalization of euthanasia or PAS. This might partly explain the differences in the results of the present and previous studies [1, 2].

According to this study, Finnish physicians do not consider withdrawal of life-supporting treatments reprehensible. Although there was a statistically significant change between the study years, the absolute difference was only 4 mm. Our results are in line with the results from a large, international study by Löfmark et al. where 72–86% of the physicians surveyed reported experiencing foregoing life-supporting treatment and only 1–6% reported never being willing to do so [27].

Knowledge about the benefits of palliative care has grown in recent decades, and it is considered a part of everyday care in life-threatening illnesses [10, 28, 29]. Therefore, it is not surprising that almost all of the respondents in 2015 considered palliative care as a way of enabling a good death. However, in another study only 51–70% of physicians believed that palliative care was able to prevent the need for euthanasia and PAS [22].

Although advanced care planning has shown a positive impact on the quality of end-of-life care [30, 31], the prevalence of advance directives varies largely. In the United States, the prevalence of advance directives seems to have increased from approximately 10% up to 21–55% among the elderly in the last 10 years [32, 33], while a Finnish study from 2004 showed that only 12% of the home-dwelling elderly had a living will [34]. In our study, physicians found the advance directives of the patients now more helpful than they were in 1999, but having an advance directive of their own was still uncommon among doctors in 2015 even though they were older and more experienced than in 1999. This finding might reflect a division between personal life values and experiences in clinical work.

In general, physicians accepted potentially lethal morphine dosing more frequently now than in 1999, although approximately two-thirds of the doctors were still unwilling to provide this. This result might be due to actual acceptance of hastening death at the end-of-life, better knowledge regarding the use of opioids or both. It is now known that clinically relevant respiratory failure is not a problem when opioids are titrated against cancer pain [35]. Since 1999, there has been growing evidence that the use of opioids for symptom control in advanced diseases has no effect on survival and even high doses of opioids do not seem to shorten life during end-of-life care [20, 21]. In a study conducted in the Netherlands, physicians administered similar dosages of opioids in 1995, 2001 and 2005; however, compared with previous years, in 2005, they thought that life was shortened by opioids or their intension was to hasten death by administering opioids less frequently [16]. On the other hand, high doses of opioids do cause respiratory depression [17–19], and the potential of opioid to hasten death during end-of-life care is almost impossible to study with prospective randomized trials. Although we did not ask the intention behind physicians’ willingness to increase the morphine dose, it was clearly stated in the patient scenario that increasing the dose might lead to the patient’s death. Oncologists were most reluctant to provide the dose increase, and their opinion did not change between 1999 and 2015. However, they were probably the most familiar with the influences of opioids in clinical practice as well as the studies on this issue. Our results reflect that surgeons, internists and GPs have become increasingly willing to hasten death according to a patient’s wishes today than they were 16 years ago, although improved knowledge on the low risk of using opioids during end-of-life care probably influenced our results.

Our results are in line with the study by Miccinesi et al. in which oncologists were the least in favour of using lethal drugs [22]. In our study, the difference between oncologists and other physician groups remained in the results of the logistic regression analysis. These findings might be observed because oncologists take care of these patients on a more regular basis and are perhaps aware that a patient’s wish to hasten death does not always imply a genuine wish to die, but might be the result of overwhelming emotional suffering [36], which could be relieved by therapy.

Religion has been confirmed to have a tremendous effect on end-of-life decisions and attitudes towards euthanasia and PAS [1, 22, 27]. In the present study, faith in God was also found to decrease physicians’ willingness to administer potentially lethal morphine dose. The number of physicians who had faith in God is lower in the present survey than in 1999, which might be one reason for the increasing support for euthanasia and hastened death. In a previously mentioned study, Löfmark et al. concluded that a non-religious philosophy of life increased physicians’ willingness to perform euthanasia and PAS, possibly by emphasizing patient autonomy [27]. Advance directives were relatively uncommon among physicians, but having one seemed to increase the willingness to double the morphine dose; however, the influence of advance directives did not quite reach statistical significance in our logistic regression analysis. To our knowledge, the influence of doctors’ own advance directives on end-of-life decisions has not been previously reported.

We suggest that completing an advance directive for oneself may lead to greater acceptance of death, even if this is hastened in a situation without hope for a cure.

Male sex and older age were independently associated with physicians’ willingness to double the morphine dose. Previous studies on these factors are somewhat controversial. Females have been shown to be less supportive towards ending life without explicit request from the patient, but also to be more supportive of alleviating pain and other symptoms regardless of the possible life-shortening effects [22, 26]. In general, younger physicians accept PAS more often but are less willing to withdraw life-prolonging treatments than older physicians [2, 22, 26]. The exact reasons why age and sex are related to the tendency to administer potentially lethal morphine dose in our study remains unknown, but perhaps more experienced physicians do not believe that such a morphine dose would actually kill the patient. Furthermore, men are reported to approve of assisted death more often than women in the general population [1].

Developments in medicine have allowed many interventions for patients with very advanced diseases, but the low survival rates for cardiopulmonary resuscitation (CPR) in the cancer population have not changed [37]. Furthermore, advanced care planning, which increases the prevalence of do-not-resuscitate orders, is probably a more common practice today than in the 1990’s [31, 38]. In our study, physicians’ willingness to start CPR in a patient with very advanced cancer was relatively low and did not change over the study years, in a contrast to the more ethically difficult and complex attitude regarding hastening death. Of interest, the decision about CPR did not influence physicians’ willingness to hasten death through the dual effect of a high morphine dose. We suggest that physicians’ willingness to hasten death is mainly related to their personal attitudes and values rather than medical facts, which probably guide the decision to withhold CPR.

Finally, we should state that changes in the surrounding society, general attitudes and clinical decision-making in Finland and Europe since the 1990s might have had a substantial influence on our results. In a large international study, the use of lethal doses of drugs after the explicit request of a patient with a terminal illness and uncontrolled symptoms was accepted by 35–78% of physicians, depending on the country [22]. This large range describes the cultural influence on the difficult decision to hasten death, but the numbers are quite similar to those found in our study. Public attitudes towards assisted death have changed since the 1990s to become more permissible, which has led to legalization of assisted death in some countries and increased political support for it in Finland [1, 2]. At the same time, knowledge and awareness of palliative care have grown in Finland through national and international recommendations [39–41]. However, this has happened later than in some other European countries such as the UK [3, 4]. In addition, patient autonomy and shared decision- making in treatment-choices are increasingly emphasized as important ethical principles throughout Western countries [5, 6, 42]. Patients’ rights regarding treatment decisions were incorporated into Finnish law in 1992, and respecting the patient’s wishes is currently one of the main principles in the ethical guidelines of the Finnish Medical Association [8, 9]. This social and cultural context together with the shift from paternalism towards a more patient-centred approach in clinical decision-making probably influenced the responding physicians’ considerations on the reprehensibility of hastened death and their willingness to comply with patients’ requests in the ethically complex situation in our study [6, 43].

### Strengths and limitations of the study

Limitations of this study need to be acknowledged. Our response rate (56%) is a limitation even though our study population is large and the response rate is higher than that in many recent studies [44–46]. The study population is also a representative sample, as it reflects the overall distribution of specialities and gender among Finnish physicians [47]. The follow-up period is long enough to detect relevant changes in attitudes and decision-making. Answers to the hypothetical scenarios might differ from the decisions made in clinical practice, but these questions are difficult to study in real life situations.

## Conclusions

Considering a hypothetical case scenario, Finnish physicians accepted the risk of hastening death more often in 2015 than in 1999. The specialty of the physician, gender, and faith in God strongly influenced their acceptance to this practice. Oncologists were the most reluctant of all the specialists studied to hasten death. Euthanasia, but not assisted suicide, was considered slightly less reprehensible in 2015. Relieving suffering, while considering the justification to hasten death, is a complex ethical question. Therefore, both training in medical ethics and medicine are needed for high quality end-of-life care.

## Abbreviations

CPR: Cardiopulmonary resuscitation
GP: General practitioner
ICU: Intensive care unit
IQR: Interquartile range
PAS: Physician-assisted suicide
UK: the United Kingdom
VAS: Visual analogue scale
